# Prevalence of *Batrachochytrium dendrobatidis* in *Xenopus* Collected in Africa (1871–2000) and in California (2001–2010)

**DOI:** 10.1371/journal.pone.0063791

**Published:** 2013-05-15

**Authors:** Vance T. Vredenburg, Stephen A. Felt, Erica C. Morgan, Samuel V. G. McNally, Sabrina Wilson, Sherril L. Green

**Affiliations:** 1 Department of Biology, San Francisco State University, San Francisco, California, United States of America; 2 California Academy of Sciences, San Francisco, California, United States of America; 3 Museum of Vertebrate Zoology Berkeley, Berkeley, California, United States of America; 4 Department of Comparative Medicine, Stanford University, Stanford, California, United States of America; Smithsonian’s National Zoological Park, United States of America

## Abstract

International trade of the invasive South African clawed frog (*Xenopus laevis*), a subclinical carrier of the fungal pathogen *Batrachochytrium dendrobatis (Bd*) has been proposed as a major means of introduction of *Bd* into naïve, susceptible amphibian populations. The historical presence of *Bd* in the indigenous African population of *Xenopus* is well documented. However, there are no reports documenting the presence of *Bd* in wild *Xenopus* populations in the US, particularly in California where introduced populations are well-established after intentional or accidental release. In this report, a survey was conducted on 178 archived specimens of 6 species of *Xenopus* collected in Africa from 1871–2000 and on 23 archived specimens (all wild-caught *Xenopus laevis*) collected in California, USA between 2001 and 2010. The overall prevalence rate of *Bd* in the tested Xenopus was 2.8%. The earliest positive specimen was *X. borealis* collected in Kenya in 1934. The overall prevalence of *Bd* in the *X. laevis* collected in California was 13% with 2 positive specimens from 2001 and one positive specimen from 2003. The positive *Xenopus* (3/23) collected in California were collected in 2001 (2/3) and 2003 (1/3). These data document the presence of *Bd-*infected wild *Xenopus laevis* in California. The findings reported here support the prevailing hypothesis that *Bd* was present as a stable, endemic infection in *Xenopus* populations in Africa prior to their worldwide distribution likely via international live-amphibian trade.

## Introduction

The appearance of the amphibian chytrid *Batrachochytrium dendrobatis (Bd)*, a fungal pathogen and the causative agent of chytridiomycosis has caused amphibian deaths and population declines worldwide [1]–[3]. This highly transmissible fungus has led to the recent decline or extinction of approximately 200 species of frogs [4]. A retrospective study of museum specimens performed by Weldon and colleagues [5] identified *Xenopus laevis*, the South African clawed frog, as an asymptomatic but *Bd*-transmitting species responsible for the pathogen’s global spread. In that study, infected *Xenopus* were identified in specimens collected as early as 1938 and another study found an infected *Xenopus* collected from Cameroon from 1933 [6]. Global distribution of *Xenopus* via the pet and zoo exhibit trade was well-established by the late 1900’s and by 1940, with the discovery that injection of urine from a pregnant woman caused egg-laying in *Xenopus*, the frogs were widely used in hospital laboratories world-wide as a means of detecting human pregnancy [7]. This practice persisted through the 1970’s. Though no longer used for pregnancy testing, *Xenopus* have since become a major animal model in biomedical and basic science research [8]. Significant populations of *Xenopus* (largely *X. laevis* and to a lesser extent, *X. tropicalis*) are currently housed in research laboratories.

Global trade of *Bd* infected *Xenopus* and their release into the wild (either accidentally or intentionally) may have contributed to the present *Bd* epidemic, and may be one of the major means by which the pathogen was introduced into the United States. There is currently a US-based movement to limit the importation, trade and transportation of this species [9]. However, to date there is no report documenting *Bd* infection in wild caught *X. laevis collected in the US, or from California in particular,* a geographic location well- known to be relatively recently invaded [1]–[3],[10].

In this report, we determined the prevalence of *Bd* in archival *Xenopus* specimens from the herpetological collection at the California Academy of Sciences (CAS), one of the oldest and largest collections in North America. This collection includes specimens from 6 different *Xenopus* species (including *X. laevis*). The samples were collected in the wild from southern, eastern, and western Africa, as well as from established introduced populations in California.

## Materials and Methods

A retrospective study was conducted on the entire collection of 201 archived specimens (collected between 1901 and 2001) of the genus *Xenopus* housed in the California Academy of Sciences. Specimens archived in the CAS were collected by several groups and were not collected for the purpose of disease surveillance. The specimens examined from this archive were collected mainly from Kenya, Uganda, and a variety of other African nations and from Los Angeles, San Diego, and San Francisco County in California, USA. The frog specimens, most preserved in ethanol, were sampled using swabbing techniques and PCR as previously described [11]. Briefly, the skin of each of the 201 preserved amphibians was swabbed with a MW100 sterile cotton-tipped swab. Each specimen was swabbed a total of 30 times: 10 strokes each along each side of the ventral surfaces (including abdomen pelvis, and thighs), and 5 strokes on each hind foot webbing. To prevent possible cross contamination from multiple specimens cohabiting in a single jar, each specimen was rinsed with 70% ethanol prior to swabbing, and a new pair of disposable gloves was used for each specimen. Swabs were stored in 1.5 mL microcentrifuge vials and refrigerated at 4°C until extraction. Prior to extraction, swab vials were placed in a SpinVac for 15–20 min or under a fume hood for ∼1 hr to evaporate residual ethanol. Swabs were extracted with Prepman Ultra, and extractions diluted 1∶10 in 0.25×TE Buffer. Presence of *Bd* was determined using a real-time PCR (ABI 7300) assay for *Bd* according to methods described by Boyle *et al* [12], and revised for museum specimens as specified in Cheng *et al* [11]. Samples were run in triplicate along with negative controls (H_2_0, TE Buffer) and positive standards at dilutions of 100, 10, 1.0, and 0.1.

## Results

Overall, prevalence of *Bd* in the CAS archived *Xenopus* specimens (African and Californian) was 4.0% (8 positives out of 201 specimens) (Table 1). The 8 positive samples were positive in all three of the triplicate runs, but the genomic equivalents were low (range 0.01–1.3 GE). Of the 6 different species of *Xenopus* archived from Africa, most of the positive specimens were *X. laevis* (6/8), but positives were also found in *X. borealis* (Table 1). The prevalence of *Bd* infection across all African *Xenopus* specimens in the CAS collection was 2.8% (Table 2). Infected frogs were present in Kenya and Uganda (Table 2). The earliest date for a positive specimen was 1934 (*X. borealis* specimen collected in Kenya; Table 3). None of the positive specimens were co-housed in the same jar.

**Table 1:** Prevalence of *Batrachochytrium dendrobatidis* in all *Xenopus* specimens tested in this study and archived at the California Academy of Sciences.

Species	No. examined	No. positive	% positive (95%^a^CI)
*X. laevis*	145	6	4.1 (1.5,8.8)
*X. borealis*	3	2	66.7(9.4, 99.2)
*X. fraseri*	2	0	0 (0, 84.2)
*X. muelleri*	11	0	0 (0, 28.5)
*X. tropicalis*	2	0	0 (0, 84.2)
*X. wittei*	38	0	0 (0, 9.3)
Total	201	8	4.0 (1.7, 7.7)

a CI = Bayesian Credible Interval

**Table 2:** Prevalence of *Batrachochytrium dendrobatidis* in archived *Xenopus*, by location.

Location	No. examined	No. positive	% positive (95%^a^CI)
Botswana	1	0	0 (0, 97.5)
Ghana	2	0	0 (0, 84.2)
Kenya	42	3	7.1 (1.5, 19.5)
Namibia	4	0	0 (0, 60.2)
Nigeria	1	0	0 (0, 97.5)
Rwanda	4	0	0 (0, 60.2)
South Africa	4	0	0 (0, 60.2)
Tanzania	10	0	0 (0, 30.9)
Uganda	88	2	2.3 (0.3, 8.0)
Zaire	9	0	0 (0, 33.6)
Zambia	9	0	0 (0, 33.6)
Unspecified, Africa	4	0	0 (0, 60.2)
USA, San Francisco, California	3	1	33.3 (0.8, 90.6)
USA, San Diego, California	8	2	25 (3.2, 65.1)
USA, Los Angeles, California	12	0	0 (0, 26.5)
Total	201	8	4.0 (1.7, 7.7)

a CI = Bayesian Credible Interval.

**Table 3:** Prevalence of *Batrachochytrium dendrobatidis* in archived *Xenopus* by time intervals, specimens collected in various countries in Africa and North America.

Time interval	No. examined	No. positive	% positive (95%^a^CI)
1871–1940^b^	18	2	11 (1.9, 30)
1941–1950	4	0	0 (0, 45)
1951–1960	28	0	0 (0, 9.8)
1961–1970	4	0	0 (0, 45)
1971–1980	40	1	2.5 (0,11.1)
1981–1990	17	0	0 (0, 15.3)
1991–2000	65	2	3 (0.4, 9.4)
1991–2000*	12	0	0 (0, 20.5)
2001–2010	2	0	0 (0, 63.1)
2001–2010*	11	3	27.2 (8.2, 54.8)
Total	201	8	4.0 (1.7, 7.7)

a CI = Bayesian Credible Interval;

b earliest positive specimen collected in 1934, *X. Borealis,* in Kenya,

* samples collected in California, USA.

The overall prevalence of *Bd* in *Xenopus* archived from California was 13% (3/23) (Table 2). The earliest positive specimens were from two *Xenopus leavis* collected in San Diego County in 2001 (CAS 220090, CAS 220098) followed by one positive *X. leavis* collected in San Francisco County in 2003 (CAS 244036). These specimens are housed in jars grouped by collection date and site.

## Discussion

This study has provided additional data for some of the earliest known positive cases *Bd-*infected amphibians in the wild: the sample reported here collected in 1934, positives collected in 1933[6], and 1938 [5], all which were specimens collected in Africa. Here we also report for the first time the presence of *Bd* infection in established introduced populations of *Xenopus* in California, an area that has experienced *Bd*-epizootic events associated with mass die offs and population extinctions of native frog species [1]–[3].

The overall prevalence of *Bd* infection in CAS archived African *Xenopus* of 2.8%, is remarkably close to the overall prevalence previously reported by Weldon (2.7%) [5]. Notably, though the sample size is small, the overall prevalence of *Bd* in *Xenopus* collected from California was relatively high: 13% (time interval 2001–2010) but is consistent with the prevalence rate reported in by Ouellet *et al* [10], who surveyed archival amphibian specimens collected in Canada and the United States between 1895–2001. *Xenopus* were not amongst the specimens surveyed from that collection, but the earliest positive infections dated from the 1960’s and were found in a native North American anuran species, *Rana clamitans*. The presence of wild *Xenopus* populations in California was first documented the 1970’s; these populations are thought to have been established via release or escape from pet shops and hobbyists, from museum and zoo exhibits, or from hospital or research laboratories [13]–[16]. The findings reported here provide temporal evidence that *Bd*-infected *Xenopus* were present in California at least as far back as 2001. This supports the epidemic pathogen hypothesis in California, i.e. that *Bd*-positive *Xenopus,* a non-native, invasive species imported from Africa and released in California are one possible means of spreading *Bd* to naïve amphibian hosts.

Eleven states (AZ, CA, HI, MN, NV, NJ, NC, OR, UT, VA and WA) in the U.S. currently restrict the importation of *Xenopus*, either by necessitating special permit requirements for research laboratories and exhibitors, and/or by not allowing this species to be sold as pets [9]. The U.S. Department of Interior’s Fish and Wildlife Service Agency is currently reviewing a petition to list all live amphibians and their eggs under the Lacy Act (thus restricting their importation and intra- and interstate transport) and determining this species as “injurious” unless certified as free of *Bd*, the amphibian chytrid fungus [9]. Although *Xenopus* are no longer used for pregnancy testing, the impact of additional regulations restricting the trade of *Xenopus* would be significant, particularly to basic and biomedical research. While the exact numbers of *Xenopus laevis* used in research world-wide are difficult to determine, since 1970 to the present, there have been >25,000 research reports using *Xenopus* [17].

Quellet et al [10], reported *Bd* positive anurans in Quebec, Canada in 1961. It is not known if *Bd* was present in California before *Xenopus* arrived, however, based on the results reported here, it does appear that wild *Bd-*infected *Xenopus* were present in California at least 12 years ago and that *Xenopus* may be, in part a means of spread of this fungus to susceptible native amphibians. Another potential source of the spread of *Bd* in California is the American Bullfrog (*Rana catesbeiana*) which was introduced to the US West Coast as a food source in the 1920’s. This species has been widely implicated in the spread of *Bd* and studies are currently underway to determine if archival specimens from this species are *Bd* positive.

Our results indicate that *Bd*-infected *Xenopus* were present in California; however, no attempt was made to sequence the material from these archival specimens. While the PCR amplification curves were normal, the genomic equivalent values were very low (0.0075, 0.01,.001), which is typical of results using this *Bd* PCR assay on museum specimens [11]. Thus we cannot at this time ascertain if the *Bd* material identified in these specimens could be an endemic *Bd* strain, or a more virulent introduced pathogenic strain or even a new sexual hybrid [18], as has been proposed to cause the recent massive amphibian die offs. Evidence was recently reported for a hypervirulent strain of *Bd* on introduced *Rana catesbeiana* [18] alongside other less virulent strains on native fauna, and this makes it harder to interpret our historical findings. The question remains: Which *Bd* strain(s) did we discover on the historical specimens reported here and did it differ in Africa from those we discovered in California? Our study cannot address this issue. Studies that use the *Bd* qPCR assay [12] should be aware that different strains of *Bd* may yield different infection intensities based on variable ITS copy number by strain [19]. Studies that employ the *Bd* assay for testing museum specimens are less likely to be affected by this, however, because there is less emphasis on infection intensities and more on simple positive negative results [11]. Additionally, there are potential sources of contamination that we could not control. For example, contemporary field measures to prevent cross contamination between specimens cannot be applied retrospectively to specimens collected in the past. When working with specimens, it is not possible to know the exact history of how each specimen was stored since the day it was collected. Many specimens, for example, do not have accompanying field notes and the only data available are those attached on the tags attached to the specimens. Future studies may be able to use new technologies to recover sequence data from museum swab PCR assays with very little recoverable *Bd* DNA, however we believe that retrospective Bd-assays on amphibian specimens provide an important basis for our understanding of the epidemiology of this disease. If wild *Xenopus* appeared in California decades ago, during the 1970s as first reported [14],[15] then by strong inference and supported by the findings reported here, introduction of *Bd* into this geographical area could have occurred in part via *Bd* infected *Xenopus laevis*.
